# The Fish Feed Sector in Kenya, Uganda, Tanzania, and Rwanda: Current Status, Challenges, and Strategies for Improvement—A Comprehensive Review

**DOI:** 10.1155/2024/8484451

**Published:** 2024-09-26

**Authors:** Jonathan Munguti, Mavindu Muthoka, Mercy Chepkirui, Domitila Kyule, Kevin Obiero, Erick Ogello, Nazael A. Madalla, Gerald Kwikiriza

**Affiliations:** ^1^ National Aquaculture Research Development and Training Center (NARDTC) Kenya Marine and Fisheries Research Institute (KMFRI), Sagana, Kenya; ^2^ Department of Animal and Fisheries Sciences Maseno University, Maseno, Kenya; ^3^ Kegati Aquaculture Research Center Kenya Marine and Fisheries Research Institute (KMFRI), Kisii, Kenya; ^4^ Sangoro Aquaculture Research Center Kenya Marine and Fisheries Research Institute (KMFRI), Pap-Onditi, Kenya; ^5^ Department of Animal Aquaculture and Range Sciences Sokoine University of Agriculture, Morogoro, Tanzania; ^6^ Kwchweano Zonal Agricultural Research and Development Institute National Agriculture Research Organization (NARO), Kabale, Uganda

**Keywords:** aquaculture production, East Africa, fish feed, policy reform

## Abstract

This review paper provides an in-depth analysis of the current status, challenges, and strategies for improvement within the fish feed industry in East Africa, focusing on Kenya, Uganda, Tanzania, and Rwanda. Aquaculture production in these countries is experiencing steady growth, driven by increasing demand for fish and fish products for both nutritional and economic purposes. Despite the market facilitating the transition from extensive to semi-intensive and moderately intensive farming systems across the four countries, the sector's progress is hampered by a lack of sustainable, locally produced, high-quality, and cost-effective fish feeds tailored to different developmental stages of fish. Despite the evident need, there is a notable scarcity of comprehensive reviews addressing the regional perspective of fish feed due to heightened cross-border trade driven by the soaring demand and increased installation of cages in Lake Victoria, as well as in inland dams and reservoirs. This paper addresses critical challenges, such as regional scarcity and limited access to quality feed ingredients, regulatory obstacles, insufficient quality control measures, infrastructure constraints, and a lack of awareness and understanding of feed management and formulation. To overcome these challenges, the paper recommends fostering collaboration to establish a robust regional fish feed supply chain, investing in research and development initiatives, advocating for policy reforms and regulatory support, and compliance with East African Community quality standards for fish feed. Moreover, there is an urgent need to enhance human resource capacity through training and extension services, promote public investment support, strengthen sector institutions and industry associations, conduct training and awareness programs for feed providers, and improve storage facilities to maintain feed quality. The paper provides policymakers with valuable insights to inform targeted interventions that will catalyze positive transformation within the fish feed industry in East Africa.

## 1. Introduction

Africa has approximately 1.5 billion people, about 18% of the world's population. Projections indicate that this number could rise to 2.4 billion by 2050 [1]. Meeting the nutritional needs of such a large population presents a significant challenge for Africa's food systems [2]. The continent faces numerous developmental challenges, including chronic food insecurity and widespread poverty, which are critical for achieving sustainable development goals [3]. Fish and other aquatic foods are vital in Africa's food systems. They are indispensable for creating livelihoods, generating income, and supplying essential micronutrients, especially for women and children. Although fisheries and aquaculture are critical, there is still a significant gap between the fish supply and consumer demand in East Africa [4].

Among East African countries, per capita fish consumption is about 5–6 kg, which is low compared to the continental average of 10 kg per person per year and the global average of 20.5 kg per person per year [5]. For instance, Rwanda's per capita fish consumption is merely 2.3 kg, significantly lower than that of its neighbors, with Kenya at 4.7 kg per person per year, Tanzania at 8 kg per person per year, and Uganda at 10 kg per person per year [4]. The lower fish consumption in these countries has been attributed to many factors, including lower fish supplies due to declining capture fisheries. To address this challenge, fish traders, for instance, in Kenya, are importing fish from China to bridge the demand gap.

Over the past few years, there has been minimal to no growth in global capture fisheries [6]. However, aquaculture in Africa has seen substantial expansion. Aquaculture now represents 16%–18% of Africa's total fish production and provides more than half of the fish consumed on the continent [7]. In 2020, global aquaculture production reached a record high of 122.6 million tonnes, with inland farmed waters contributing around 54.4 million tonnes. Data show that the development of aquaculture in Africa surpasses the global average [8]. This rapid expansion has prompted the East African Community (EAC), a regional economic body, to focus on boosting investments in aquaculture to meet the increasing demand for fish.

In Kenya, the government seeks to raise aquaculture production from 31,000 to 100,000 tonnes in the medium term and 400,000 tonnes by 2030, while Uganda aims to ramp up its aquaculture output to 1,000,000 tonnes by 2030 [9]. The increasing reliance on aquaculture as a main source of nutritious aquatic food has led to a higher demand for high-quality fish feeds. To support this shift, it is crucial to develop sustainable milling businesses and establish robust partnerships with international organizations and development partners. These collaborations play a vital role in maintaining a steady supply of aquafeed and its necessary components [10].

However, the aquaculture sector's performance has been suboptimal, primarily due to the lack of locally produced, high-quality, and affordable fish feeds designed for the various developmental stages of fish, as well as the necessary raw materials [11]. These raw materials include plant-based ingredients like corn, rice, rice bran, wheat bran, sunflower cake, and soybean seeds, as well as animal-based ingredients such as fish meal, shrimp meal, blood meal, and poultry byproduct meal. In Kenya, around 7000 tonnes of aquafeed are imported annually, mainly from small-scale fish feed manufacturers in East Africa and other countries [12]. The use of commercial fish feeds in Tanzania has increased fivefold from 1182 tonnes in 2021 to 6211 tonnes in 2023, with more than 50% imported [13]. Despite the surge in demand for commercial fish feed, their prices have remained high, compelling many farmers to formulate their feeds or resort to noncomplete feeds, resulting in suboptimal fish production output. Many scholars acknowledge that aquaculture growth is closely tied to high-quality feeds that meet the nutritional requirements of farmed fish [12, 14]. Therefore, locally produced fish feed is crucial in reducing production costs, making fish farming more attractive to both private and commercial investors, and increasing fish production, particularly in East Africa.

Despite the burgeoning potential of the aquaculture industry in East African countries, fish feed suppliers and investors have been reluctant to invest in the industry due to the relatively nascent stage of development compared to more established markets globally. Fish feed suppliers may see higher risks in emerging aquaculture sectors like Uganda or Kenya, where market demand, profitability, and regulatory frameworks are less predictable. As a result, these investors often allocate their capital to more mature and stable markets with established infrastructure, transparent regulatory environments, and proven track records of success. Nonetheless, this presents a unique opportunity for entrepreneurs and investors to be pioneers in an emerging market with vast potential for growth and innovation.

Fish feeds constitute approximately 70% of the expenses for fish farmers in both semi-intensive and intensive culture systems, with protein emerging as the costliest macronutrient [15]. In semi-intensive tilapia farming, where ponds receive heavy fertilization, natural food organisms contribute substantially to the necessary nutrients for fish growth [16]. However, small-scale farmers often find commercial feed prices prohibitive and resort to using either farm-compounded or low-quality commercial feeds to supplement natural food and maximize yields in the production systems. With this practice, the production and productivity of aquaculture remain low. For significant growth and realization of its potential, the development of the East African fish feed industry necessitates redirection [17], which can only be done if there is a clear understanding of the industry's challenges and clear strategies for solving the challenges.

Despite the evident need, there is a notable scarcity of comprehensive reviews addressing the regional perspective of fish feed due to heightened cross-border trade driven by the soaring demand and increased installation of cages in Lake Victoria, as well as in inland dams and reservoirs. The objective of this review is to unearth valuable insights into the obstacles hindering the industry's sustainable development and identify strategic avenues for improvement. These insights can inform national governments in formulating policies to strengthen the fish feed industry, foster its growth, and enhance its contribution to food security and economic development. Therefore, investing in research to elucidate these challenges and devise effective strategies for improvement is beneficial and crucial for enabling policymakers to enact informed and targeted interventions to catalyze positive change in the industry.

## 2. Methodology

A systematic literature review was used to explore the fish feed industry's status, challenges, and strategies in Kenya, Uganda, Tanzania, and Rwanda. The literature search was performed in several academic databases to identify all relevant studies. These databases were chosen among others such as Web of Science (http://www.isiknowledge.com), Scopus (https://www.scopus.com/), Google Scholar (https://scholar.google.com/), and Semantic Scholar (https://www.semanticscholar.org/). Included databases had to represent broad academic discipline coverage, regional diversity, English language publication, and the capacity to use advanced searching capabilities.

The search terms were determined based on discussions with relevant stakeholders to ensure that all peer-reviewed and gray literature on the fish feed industry in East Africa was captured. The search framework used key terms in the title search field, including “Aquaculture,” “Fish feed,” “Industry,” “East Africa,” “Kenya,” “Uganda,” “Tanzania,” “Rwanda,” “Challenges,” and “Strategies.” The search terms were adopted in the titles, abstracts, and keywords parts of the databases to complete the search as we expected. The search eligibility was refined using very specific inclusion and exclusion criteria to ensure that only the most authentic and most accurate results were included in our list. The six inclusion criteria included (i) published from January 2003 to May 2024, (ii) fish feed industry and aquaculture, (iv) related topics, (v) of East Africa, and (vi) written in English articles. Exclusion criteria included articles not available in full text, studies unrelated to the fish feed industry or aquaculture, or those not meeting the relevance criteria (e.g., findings on context not relevant to East Africa).

**Figure 1:**
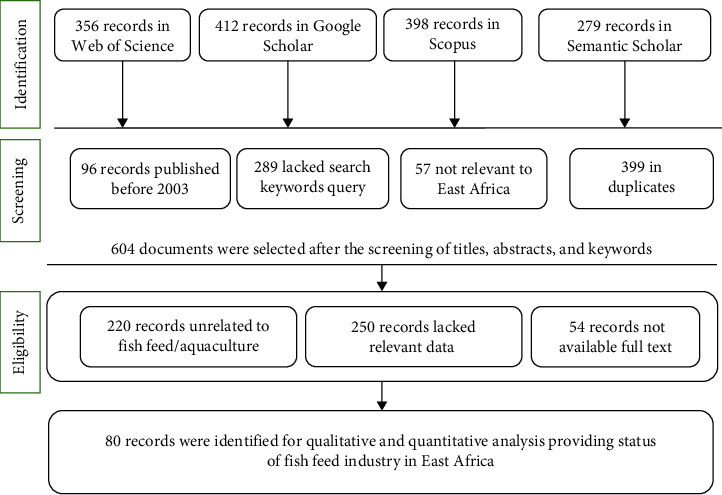
PRISMA statement process undertaken for the selection of relevant articles.

To create an article database, the Preferred Reporting Items for Systematic Reviews and Meta-Analyses (PRISMA) statement (Figure 1) was utilized [18]. The PRISMA statement follows a structured process that includes identification, screening, eligibility, and inclusion phases. In the identification phase, we searched four databases and found a total of 1445 articles: 356 records from Web of Science, 412 from Google Scholar, 398 from Scopus, and 279 from Semantic Scholar. During the screening, we eliminated 96 articles published before 2003, 289 records lacking key search queries, 57 articles irrelevant to East Africa, and 399 duplicate records. This left us with 604 records to move to the eligibility phase. In the eligibility phase, we reviewed the 604 remaining records and excluded 524 based on the following criteria: 220 records were unrelated to fish feed and aquaculture, 250 records lacked relevant data, and 54 records were not available in full text. After this rigorous process, 80 articles were deemed suitable for full-text reading and further analysis. This structured and transparent approach, guided by the PRISMA statement, ensured a thorough and unbiased selection of articles relevant to the fish feed industry and aquaculture in East Africa.

## 3. Overview of the Current Status of the Fish Feed Industry in East Africa

Over the past decade, the fish feed industry in East Africa witnessed substantial growth alongside the expansion of aquaculture in the region [4]. This surge in aquaculture production, notably observed in Kenya, Uganda, Tanzania, and Rwanda, can be attributed to a transition from extensive to semi-intensive and intensive farming systems [7]. Among the intensive production systems are cages and raceways, which require more significant quantities of feed, thus driving up the demand. Based on a recent survey in 2022, Lake Victoria, Kenya, has 5242 cages [19], increasing the demand for fish feeds. Before 2010, feed mills primarily focused on producing feed for land animals and only catered to fish feed upon specific requests from farmers, owing to the limited demand [20]. To address the growing demand, efforts were made to bolster local feed production capacity, including establishing new feed mills and increasing feed imports to cater to the higher demand. Despite these efforts, major aquaculture investors in East Africa continue to rely heavily on imported feeds due to superior quality, cost-effectiveness, insufficient local production coupled with high investment costs, and competition for ingredients from other animal farming sectors [21].

In response to the demand, numerous distributors and fish feed manufacturers have emerged in the region, importing branded fish feeds while some make their own. Uganda's most prominent fish feed manufacturers and distributors include Ugachick, Kafika Animal Feeds, Ranaan Fish Feeds, Koudijs Uganda, and Victoria company. These fish feed manufacturers produce an estimated 75,000 tonnes of feed annually. However, this amount is significantly lower than the 120 million tonnes needed to adequately support Uganda's aquaculture production, which stands at approximately 111,023 tonnes annually [22]. This gap in feed demand necessitated larger aquaculture farms to import feed from other countries. Recent import data from Volza indicates that Uganda primarily imports fish feed from Zambia, Egypt, and Brazil, while Kenya sources from Zambia, Egypt, and the Netherlands. Tanzania's imports mainly come from Zambia, Vietnam, Saudi Arabia, Uganda, Kenya, and the Netherlands, while Rwanda relies on imports from India, Belgium, and the Netherlands [23].

Additionally, some large farms have forged direct partnerships with reliable international manufacturers that produce quality feed. In Kenya, some partnerships have been initiated, including Tunga Nutrition, a collaboration between Skretting and the Unga Group, which established a fish feed processing plant with an initial production capacity of 25,000 tonnes annually. Another recent collaboration involves Victory Farms, Maxim Agri Holdings, and Gatsby Africa, resulting in the SamakGro Fish Feed Factory in Naivasha [24]. As the industry continues to expand, there has been a notable improvement in local feed manufacturing, marked by significant investments in milling facilities and tax waivers for most investors interested in the feed industry [24]. For instance, local fish feed production in Tanzania has increased almost fourfold from 710 tonnes in 2021 to 3,455.4 tonnes in 2024 [13]. In Uganda, the importation of plant and machinery is zero-rated, meaning no import duties are applied. Additionally, value-added tax (VAT) on these imports is deferred, and the withholding tax is set at 6%, provided the cost of the plant and machinery exceeds US $22,500. Since 2015, the governments of Kenya, Tanzania, Uganda, and Rwanda have progressively eliminated taxes on both animal feeds and the premixed raw materials used in their production [25]. This policy change aims to enhance access to animal products and stimulate the feed production industry across East Africa.

However, most locally produced fish feeds are too costly for small-scale farmers (Table 1) [12]. Subsequently, many small-scale farmers opt for homemade feeds, often lacking quality and essential minerals, to increase growth rates. This preference stems from the intense competition for raw materials in East African countries, where resources are in high demand by both the human and livestock sectors, yet production levels remain low [26].

For instance, Rwanda's aquafeed industry relies on diverse ingredients, with rice, wheat, and maize bran being the most commonly used [27]. Additionally, producers frequently incorporate other plant-based materials such as cassava flour, cottonseed cake, soybean meal, and sunflower cake [28]. While many of these ingredients are sourced from local markets, some manufacturers have cultivated their own crops [29]. This is a common practice among the East African countries. Many plant-based ingredients, despite their extensive use, contain high levels of indigestible organic matter, mainly in the form of insoluble plant fibers. They also frequently lack essential amino acids such as lysine, methionine, and tryptophan [30]. As a result, when animal protein is completely replaced with plant protein in formulated fish feeds, it becomes necessary to supplement with synthetic lysine and methionine.

**Figure 2:**
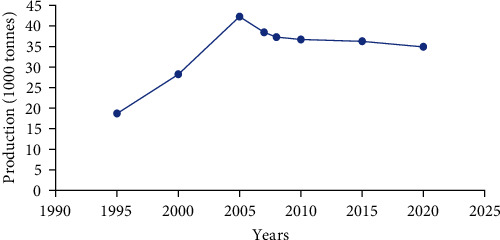
Global use and demand for fishmeal (1000 tonnes), 1995–2020 (adapted from Tacon [32]).

**Figure 3:**
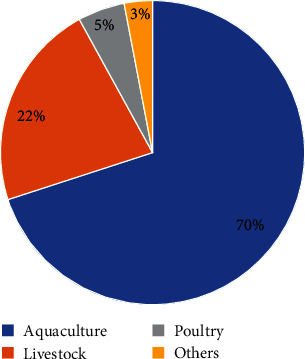
Data supplied by the Marine Ingredients Organisation [34].

On the other hand, animal protein sources, including fish meal and freshwater shrimp, are sourced from the fish landing sites, while bone meal is typically sourced from abattoirs [28]. Fish meal, blood meal, oilseed cakes, and lake shrimp are primary protein sources in aquafeeds [31]. Fish meal, in particular, is in high demand (Figure 2) due to its suitability as a protein source for most fish species. In 2017, the aquaculture sector accounted for approximately 70% of global fishmeal consumption, with livestock and poultry sectors following at 22% and 5%, respectively, while human consumption comprised 3% [33] (Figure 3).

Over time, aquaculture has decreased its dependency on fishmeal, with global consumption dropping from 4.23 million tonnes in 2005–3.49 million tonnes in 2020 [35]. In East Africa, fishmeal is primarily derived from lower-grade fish species, particularly sun-dried sardines, known locally as “omena” in Kenya, “mukene” in Uganda, and “dagaa” in Tanzania (*Rastrineobola argentea* and *Stolothrissa tanganicae*) [27]. The availability of fishmeal is often limited, and its protein content can vary significantly due to factors like season, fishing location, and handling practices, typically ranging from 40% to 60% [25, 27]. The high demand for small sardines for both fishmeal production and direct human consumption increases their cost in feed formulations, making this approach unsustainable as these fish are also a valuable food source for humans [27]. Therefore, substituting fishmeal with alternative ingredients from sustainable sources that are not intended for human consumption could lower feed costs and the price of food fish, particularly in landlocked developing countries such as Rwanda and Uganda.

Locally available animal-derived ingredients like blood meal, poultry byproducts, and fishery byproducts are cost-effective, accessible, and suitable for various aquaculture diets as alternative protein sources [27]. For instance, blood meal, which is high in protein, can be used effectively in the diets of carnivorous fish species such as catfish and tilapia. Poultry byproducts, which provide essential amino acids, are often included in the feed for omnivorous species like tilapia and carp. However, while poultry byproduct meal is palatable and high in protein, it lacks sufficient dietary methionine and lysine [36]. Each ingredient provides specific nutritional benefits tailored to the dietary needs of different cultured species, making them versatile components in aquaculture feed formulations. Combining poultry byproduct meal with other lysine-rich ingredients, such as fishery byproducts, could be a viable solution [37]. Brewer's yeast biomass, a significant byproduct of the brewing industry in Rwanda, remains underutilized despite its high crude protein content (380 g/kg DM) [27]. Since the 1990s, brewer's yeast has been effectively used in aquaculture feeds and shows promise as a substantial alternative to fishmeal protein in Nile tilapia diets [20].

In East Africa, fish oil is seldom utilized, primarily due to its limited availability and high market price, alongside the low lipid requirements of freshwater fish such as tilapia. These requirements can be met with C18 polyunsaturated fatty acids at about 1% of the diet's dry weight [38]. Instead, locally available plant oils such as sunflower oil, crude palm oil, and soybean oil serve as viable substitutes, with sunflower oil being the most prevalent [23, 27]. Additionally, commercially manufactured feeds are enhanced with essential vitamins and mineral premixes to ensure fish health and growth.

Many of the raw materials used in fish feed production, both animal and plant-based, are also primary food sources for the local population, resulting in limited availability for the animal feed industry [26]. In Kenya, it is estimated that feed manufacturers use about 450,000 tonnes of raw materials each year, with a value of approximately 6 billion Kenyan Shillings. To supplement these local resources, around 2200 tonnes of omena fishmeal are imported annually from Tanzania to support Kenya's domestic industry [39].  Table 2 presents the sources of the common fish feed ingredients used by the millers in Kenya, Uganda, Tanzania, and Rwanda. The table shows that there is a shortage of most of the suitable feed ingredients, which poses a significant challenge for the fish feed sector [39], leading to a heavy reliance on imports to meet demand.

## 4. Challenges Facing the Fish Feed Industry in Kenya, Uganda, Tanzania, and Rwanda

### 4.1. Regional Lack of Quality, Reliable, and Affordable Fish Feed and Feed Ingredients

The fish feed industry in East Africa grapples with a significant challenge regarding the availability and accessibility of quality fish feeds and feed ingredients. Despite the presence of local feed ingredients, they are often unaffordable to fish farmers, particularly for animal- and plant-based components. This price surge stems mainly from competition from other sectors like human and livestock consumption [26] and the seasonal nature of agricultural production [40]. For instance, cereals are plentiful and economical during harvest seasons but progressively become more expensive until the next harvest cycle. Additionally, the availability of raw materials fluctuates seasonally, with agricultural inputs being most abundant during harvesting times. For example, Kenya experiences two rainy seasons—long (April to July) and short (October to December)—each influencing crop planting and harvesting schedules [41].

Similarly, fish meal, derived from the locally available sardine, experiences peak availability during specific periods, such as the intermonsoon winds in March to April and November to December [41]. This species has a growing regional market where it is increasingly used as human food, serving as a relatively inexpensive protein source for economically disadvantaged groups [41]. However, due to its increased demand as human food, its availability and cost-effectiveness as an animal feed source have diminished. The heightened demand for fishmeal, in general, has driven up prices for fish feed containing fishmeal, affecting its affordability and nutritional value [41]. The fish feed market in Rwanda is underdeveloped, with limited access to high-quality feed. Most farmers use homemade or substandard feeds, resulting in poor fish health and lower yields [41].

Further, climate change has exacerbated these challenges by affecting the availability and quality of raw materials used in fish feed production [42]. Changes in temperature, precipitation patterns, and the frequency of extreme weather events have disrupted agricultural productivity, leading to fluctuations in the supply of essential feed ingredients like maize, soybean, and fishmeal. Additionally, climate change has impacted the ecosystems that provide these raw materials, reducing the overall yield and quality of crops and fish that are critical for feed production [43]. For instance, the variability in rainfall and increased temperatures have led to poor harvests, reducing the availability of maize and soybean, which are key ingredients in fish feed. This scarcity has driven up costs, making it more challenging for local feed producers to maintain a consistent supply of high-quality feed. Furthermore, the increased frequency of extreme weather events, such as floods and droughts, sometimes damages infrastructure and disrupts transportation networks, hindering the distribution of feed ingredients and finished products [44].

To meet the demand for fish feed, the EAC resorts to importing ingredients from other countries. However, these imported ingredients often come at a high cost, producing expensive formulated fish feeds [45]. This poses a significant challenge, particularly for extensive/small-scale fish farmers, who constitute over 90% of the total farming population. Moreover, the prices of imported ingredients are susceptible to volatility, dependent on fluctuating exchange rates, which further exacerbates the affordability issue. In response to the high cost of imported feeds, many fish farmers, particularly in Tanzania, have adopted a do-it-yourself approach, becoming “on-farm local feed producers.” Many farmers (80%) rely on locally available feed ingredients to supplement their fish diets [46]. However, this reliance on farm-made feeds often results in lower production rates due to a lack of expertise in feed formulation, resulting in poor quality of the farm-made feeds.

### 4.2. Regulatory Hurdles and Policy Issues

Import data from Tanzania reveal that a significant portion, approximately 74%, of fish feeds are sourced from external markets, while only 26% are domestically produced [47]. Despite ongoing efforts to bolster local production, a considerable disparity between supply and demand persists. By April 2022, Tanzania boasted six private fish feed factories, yielding 540.6 metric tonnes. However, the government concurrently issued permits to import a staggering 1615.5 metric tonnes, representing 75% of the feeds utilized within the country [48]. Despite VAT and import duty exemptions, imported feed prices remain high, rendering them unaffordable for most fish farmers. This price inflation is primarily driven by global shocks such as the COVID-19 pandemic, the Russia-Ukraine War, and disruptions in global supply chains. However, without locally produced high-quality starter and grow-out feeds, these duties inadvertently serve as formidable barriers to accessibility and affordability [49].

In Kenya, the situation is further complicated by specific import duties targeting various feed ingredients. For instance, oilseed cakes incur a 10% duty, while maize sourced outside the EAC is subjected to a staggering 50% levy. Wheat imports, on the other hand, are met with a 10% duty. Byproducts like wheat or maize bran are also taxed at 10%. Moreover, importing certain premixes attracts varying duties, ranging from 10% to exemption, but accompanied by a 16% VAT. Kenya's reliance on approximately 70 types of imported premixes further exacerbates the financial burden on the industry [50]. Beyond import duties, additional charges associated with the importation process, including import declaration fees (3.5%), railway development levy (2%), and an array of regulatory fees levied by organizations such as Kenya Bureau of Standards (KEBS), Kenya Plant Health Inspectorate Service (KEPHIS), and the Department of Veterinary Services, alongside port-related expenses like stevedoring, wharfage, clearing fees, and agency charges, significantly inflate the overall cost. These ancillary charges can collectively amount to approximately 15% of the customs value [50].

### 4.3. Uncoordinated Quality Control of Fish Feed and Raw Materials

Ensuring the quality of fish feed is paramount for food safety, given that fish is destined for human consumption [51]. Established feed companies must implement Hazard Analysis and Critical Control Point (HACCP) and good manufacturing practices (GMP) systems to be certified. However, there is a notable deficiency in comprehensive HACCP or GMP systems within the fish feed sector across the region [52]. This gap in structured quality assurance extends across supply chains, processing operations, feed formulation, and marketing/distribution channels. External auditing is almost absent, leading to minimal standards for raw materials and insufficient product labeling [53]. While some large-scale mills may follow quality protocols on paper, practical implementation often falls short. Standards and guidelines are inconsistently applied, enforced, or sustained. It appears that only larger mills making concerted efforts to comply with regulations undergo auditing, leaving the informal sector largely unregulated [45].

For example, agricultural byproducts frequently utilized as standalone fish feeds or as constituents for compound feeds encompass cereal bran such as maize, wheat, rice, and oilseed cakes like cotton, soybean, and sunflower. However, the quality of these brans varies significantly, contingent upon factors such as locality and processing methods. Take rice bran, for instance, sourced from the Mwea Rice Factory in Kenya, which historically boasted a crude protein content of around 10% [31]. Following the factory's closure, other processors emerged, yielding rice bran with reduced crude protein levels, ranging from 3% to 6% [54]. Notably, these processors often adulterate their bran with milled rice husks, diminishing protein content. In contrast, wheat bran procured from industrial processors maintains a more consistent quality, typically containing 14%–17% crude protein. Despite their potential, all these materials are commonly circulated nationwide and at the regional level for livestock feed application. However, their availability fluctuates based on seasonal, regional, and demand-driven factors.

Additionally, fish feeds available in the region vary significantly in quality. Label information often does not accurately reflect the composition, particularly regarding proteins and fats [49]. This lack of transparency poses problems for fish farmers who are uncertain about feed quality and creates barriers for most companies supplying high-quality feeds to East Africa. Furthermore, fish feeds from small-scale manufacturers often lack rigorous quality monitoring, resulting in a majority of feeds being of poor quality. For example, in Rwanda, feed quality is affected by both raw material quality and processing methods [55]. Fish meal from *Haplochromis* contains excessive fiber, reducing pellet buoyancy, while aflatoxin contamination in stored maize poses a common threat [22]. Only three food processing companies in Rwanda possess the equipment to analyze this toxin, with a single FOSS 2500 analyzer available at PEAL (Poultry lab), a private company in Bugesera, for routine quality control. However, at 5000 RWF ($5.89) per sample, the analysis cost is prohibitively expensive for small-scale fish farmers. Another analyzer at a public institution, the RSB, is more accurate but costly, US $200 per sample, with a 2-week wait for results.

Feed formulation lacks efficiency without a centralized and affordable testing facility, leading to discrepancies between stated and actual feed compositions. Consequently, fish farm performance suffers, and trust in feed manufacturers wanes [48, 56]. In response to these issues, the EAC Member States, through the Lake Victoria Fisheries Organization (LVFO), have developed the “Regional Guidelines for the Certification of Fish Seed and Fish Feed” to standardize and enhance quality control measures across the region [57]. Despite these efforts, many East African countries' fragmented and ineffective regulatory landscape, compounded by limited financial resources, inhibits the establishment of integrated legislative and enforcement mechanisms. Stakeholders struggle to coordinate effectively within this disjointed system, hindering industry progress.

### 4.4. Infrastructure Limitations

While local private producers drive the feed business, their uneven distribution and production activities fall short of meeting the region's aquafeed demand. Consequently, many aquaculture farmers in Uganda have resorted to producing their feed to sustain their operations [50, 51]. However, the lack of adequate technologies, electricity, and machinery on most fish farms in rural areas hinders the preparation of farm-made feeds. Hand-operated mincers further restrict the scale of fish farming operations [58]. This calls for addressing the challenge above by strengthening training on feed-making technologies.

Inadequate storage facilities for feeds and ingredients worsen the challenges faced by smallholder farms. In Uganda, fish feed ingredients often come from agroecological zones with hot and humid conditions, which can promote the growth of mycotoxigenic fungi if not stored properly. A study by Namulawa et al. [22] found that 48% of factory and 63% of farm samples of fish feed from the Lake Victoria Basin were contaminated with aflatoxin B1, with toxin levels ranging from less than 40 to more than 400 µg/kg. This contamination poses a significant risk to fish feed production and aquaculture.

This problem, often due to a lack of knowledge or resources, leads to considerable feed spoilage [59]. Many farmers in East Africa are unaware of the importance of proper feed handling, resulting in improper transportation, handling, and storage practices. For example, transporting feeds in open trucks, motorbikes, or bicycles exposes them to high moisture content, increasing the risk of fungal infection [45]. Additionally, prolonged storage in unsuitable conditions can lead to infestation by pests and rodents, further compromising feed quality and reducing fish yields [60]. Poor feed storage conditions result in nutrient losses, feed spoilage, reduced fish yields, and poor economic returns, negatively impacting the profitability of farm operations.

### 4.5. Lack of Awareness and Knowledge on Fish Feed Management

Many farmers in East Africa have a limited understanding of feed quality, management, and the nutritional requirements of fish [41]. When calculating feed rations, they often fail to feed their fish according to recommended rates and overlook factors like ambient temperature, body mass, and pond biomass [61]. Poor record-keeping further complicates matters, as farmers struggle to adjust daily rations without accurate data. Additionally, many lack the knowledge and skills to monitor and record feed utilization, hindering their ability to use feed conversion ratios (FCRs) to assess feed efficiencies. Furthermore, inadequate records on stocking rates, mortalities, and water quality make it challenging for farmers to evaluate and optimize their production systems, undermining their ability to implement effective management strategies and improve production efficiencies [45]. This lack of proper feed management skills affects profitability and erodes trust between farmers and feed millers. Farmers may wrongly attribute low profitability to substandard feeds supplied by millers, further stunting the growth potential of the fish feed industry.

### 4.6. Feed Formulation Challenges

Properly formulated feeds are crucial for successful aquaculture production, yet many feed manufacturers struggle to provide species-specific nutrition that caters to the different life stages of fish [62]. In East Africa, there is a lack of detailed information on the nutritional content of local ingredients. Consequently, most locally formulated feeds depend on international laboratory analyses of high-quality ingredients from literature, lacking sufficient local scientific research to validate their effectiveness in fish production [63]. This problem is compounded by manufacturers often ignoring the specific nutritional requirements of farmed species during feed formulation, resulting in the widespread use of inappropriate feed compositions in the region's fish farming industry.

Moreover, some farmers utilize commercial grow-out feeds with excessive protein levels or feeds intended for entirely different fish species, disregarding the established nutritional requirements of local species [45]. Although considerable research has been conducted to ascertain these requirements, dissemination of this knowledge to farm-made feed producers and small-scale manufacturers remains inadequate. Consequently, farmers often lack awareness of the nutritional needs of their target species, leading to nutrient deficiencies that hinder fish growth and health [64].

Additionally, poorly mixed farm-made feeds contribute to subpar production outcomes, although properly formulated feeds can enhance aquaculture productivity by reducing production costs [65]. Local ingredients used in feed formulation contain sufficient nutrients, presenting an opportunity for cost-effective production through farm-made feeds [46]. However, farmers face challenges in properly mixing feed ingredients to ensure a balanced supply of essential amino acids and other nutrients [41]. A study in Tanzania by Mramba and Kahindi [66] revealed that farmers using commercial feeds achieved higher fish yields than those using farm-made feeds, attributed to improper mixing of local feed ingredients and inadequate storage practices leading to nutrient degradation.

### 4.7. Financial and Subsistence Challenges

Rurangwa and Kabagambe [55] highlight the pervasive challenge fish farmers face in Rwanda and East Africa regarding the consistency of their feeding practices. Financial constraints frequently hinder farmers' ability to maintain regular feed purchases, leading to erratic feeding schedules for their fish stocks. This irregular feeding pattern impedes fish growth and compromises the quality of the fish. Furthermore, the dependence on subsidies exacerbates this issue, as farmers may become reliant on subsidized inputs rather than investing in sustainable farming practices. This reliance on external support fosters a mentality of dependency on public funds rather than fostering a mindset of entrepreneurship and self-sufficiency within the aquaculture industry.

Moreover, the sporadic availability of funds for purchasing feed contributes to a cycle of inconsistency in fish farming operations. Farmers may struggle to predict their cash flow accurately, leading to ad hoc decisions on feed purchases based on immediate financial constraints rather than long-term planning. This unpredictability in feed procurement further disrupts feeding schedules and exacerbates the challenges of achieving optimal growth rates and feed utilization efficiency in aquaculture operations [55]. Consequently, fish farmers may be caught in a cycle of financial instability, hindering their ability to invest in essential inputs for sustainable fish production.

Additionally, relying on subsidized inputs may perpetuate a culture of reliance rather than nurturing self-sufficiency and entrepreneurship in the industry. While initially intended to support small-scale farmers, subsidies can inadvertently discourage investment in improving farming practices or exploring alternative income streams. This overreliance on external support undermines the long-term sustainability of the fish farming industry, as it discourages innovation and impedes the development of resilient, self-sustaining farming enterprises.

### 4.8. Inadequate Research and Development (R&D)

Inadequate R&D is a significant challenge facing the fish feed industry in Kenya, Uganda, Tanzania, and Rwanda. This issue manifests differently across these countries but has a common theme of hindering the growth and sustainability of the aquaculture sector. In Kenya, the aquaculture sector struggles with insufficient investment in R&D to develop affordable and locally sourced fish feed alternatives. This has led to a dependency on imported feed, which is costly and occasionally unavailable. The lack of locally produced feed increases production costs and creates vulnerability to international market fluctuations. Research has indicated that the development of the Kenyan aquaculture industry is significantly hampered by several factors, including a shortage of certified quality seed and feed, the absence of a comprehensive aquaculture policy, and insufficient funding for research [45].

Uganda faces similar challenges, where the lack of R&D in the fish feed sector results in few innovations in feed formulations. This stagnates the growth of the aquaculture industry by preventing improvements that could reduce costs and enhance feed efficiency. The absence of new research and innovative practices means that Ugandan fish farmers continue to rely on outdated methods that are less efficient and more expensive, limiting the sector's potential to contribute to food security and economic development [67]. In Tanzania, limited R&D efforts result in a lack of locally tailored feed solutions that could effectively utilize available raw materials. This gap hinders the development of cost-effective and nutritious feeds essential for the aquaculture sector's sustainable growth. A comprehensive analysis of Tanzania's fish, seed, and feed value chains identified critical factors impeding aquaculture development, emphasizing the need for collaborative research and enhanced delivery of extension services to address these issues [47].

Rwanda also suffers from minimal investment in R&D for fish feed production. Without scientific advancements and innovative practices, the sector relies on outdated and inefficient feeding methods. This reliance prevents the development of more sustainable and cost-effective feed options, which are crucial for the growth of the aquaculture industry in Rwanda [68]. The overarching challenge across these countries is the need for increased investment in R&D to foster innovation in fish feed production. This would involve financial investment and establishing supportive policies and frameworks encouraging collaboration between researchers, the private sector, and government bodies.

### 4.9. Influencing Fish Consumption Patterns

Fish consumption patterns in East Africa are heavily influenced by social and cultural factors, impacting the fish feed industry [69]. Understanding these influences is crucial for developing effective strategies to enhance fish consumption and support the region's aquaculture sector growth. One prominent example is the cultural beliefs of the Maasai community, who consider fish a taboo food. This deeply ingrained belief significantly reduces the demand for fish within Maasai-populated areas, impacting the local market for fish and, consequently, the demand for fish feed. Similarly, adherents of the Seventh Day Adventist Church abstain from consuming African catfish, further influencing fish consumption patterns [70]. Given the large number of adherents to this faith in East Africa, this religious dietary restriction further limits the market for African catfish, affecting its production and the associated demand for specific fish feeds.

These cultural and religious taboos create a significant challenge for the fish feed industry. Reduced local consumption translates to lower demand for fish farming, which directly impacts the volume of fish feed required. Fish feed manufacturers must navigate these cultural landscapes to find viable markets for their products. This challenge is compounded by the need to produce feeds for species that are culturally acceptable, which may not always align with the most commercially viable or environmentally sustainable species.

## 5. Strategies for Improvement

### 5.1. Collaboration to Build a Regional Fish Feed Supply Chain

Considering aquaculture activities across the EAC countries is essential for fostering structural changes within the region's interconnected fish value chain [49]. A regional approach is paramount due to the interdependence among countries. For instance, insufficient demand for aquaculture feeds hampers investment in fish feed manufacturing, while the lack of available fish feed discourages entrepreneurs from investing in aquaculture farms. This situation persists until a critical mass of farms and fish volumes is reached. Therefore, addressing demand and supply regionally rather than at the country level becomes imperative.

A notable initiative in the aquaculture sector is the Tunga Nutrition Partnership, which includes Nutreco-managed joint ventures with Unga Group subsidiaries: Unga Farm Care (EA) Limited in Kenya and Unga Millers (U) Limited in Uganda [71]. Tunga Nutrition Kenya was established to enhance production capacity at its jointly owned fish feed plant in Nairobi, marketing the products under the Skretting and Fugo brands. Meanwhile, Tunga Nutrition Uganda aimed to transform Unga Millers' inactive flour mill in Kampala into a modern facility for producing animal feeds and concentrates, which are sold under both Trouw Nutrition's Hendrix and Unga's Fugo brands [71]. This partnership has brought substantial investment to Eastern Africa, aiding Kenya and Uganda in meeting the increasing demand for high-quality protein by boosting local fish feed production, reducing dependence on imports, and improving the availability of premium feed for fish farmers.

Another notable example is the SamakGro Fish Feed Factory in Naivasha, Kenya, a collaboration involving Victory Farms, Maxim Agri Holdings, and Gatsby Africa [72]. This facility has enhanced local production capabilities and contributed to meeting the growing demand for fish feed in the region. Similarly, Ugachick in Uganda has invested in advanced feed production technologies, positioning itself as a leading fish feed manufacturer and producing high-quality feeds that support the local aquaculture industry [73]. Their model demonstrates how investment in technology and capacity building can drive sector growth. These initiatives exemplify how collaboration to build a regional fish feed supply chain can be effective. By leveraging partnerships between local and international companies, these projects have managed to pool resources, expertise, and technology, fostering a more integrated and robust supply chain. Such collaborations ensure a consistent supply of high-quality feed across the region, addressing the demand–supply gap more efficiently than isolated efforts within individual countries. Moreover, these partnerships have facilitated knowledge transfer and innovation, improving feed quality and production methods.

Businesses involved in the aquaculture value chain cannot solely rely on the conditions within individual countries to make investment decisions. It requires a broader perspective that encompasses the entire region. Additionally, government intervention plays a crucial role in facilitating this regional approach. Governments must engage in agreements to liberalize trade across East African borders and establish independent feed quality control mechanisms at the regional level. This ensures standardized quality across borders and promotes trust among stakeholders in the aquaculture industry [49].

### 5.2. R&D Initiatives

R&D initiatives can be crucial in addressing ingredient sourcing and quality concerns in the fish feed industry. One approach is to invest in exploring alternative sources of raw materials that are more affordable and sustainable. For example, there is growing interest in utilizing insects as a source of protein and other nutrients in fish feed production [74]. Insects can be a cost-effective and environmentally friendly alternative to traditional feed ingredients like fish and soybean meal. Research focused on understanding the nutritional composition and optimal inclusion levels of insect-based ingredients can help diversify the sources of raw materials for fish feed production [75].

Another area of interest is developing feeding strategies such as mixed feeding schedules and skip days for cost reduction without compromising performance [76]. Furthermore, investing in R&D can lead to innovations in feed formulation techniques to enhance fish feed's nutritional quality and digestibility. In countries like China, where aquaculture has experienced significant growth, substantial investments have been made in research and innovation. Current efforts are focused on exploring nutrient metabolism and related signaling pathways to achieve precise nutrient regulation and meet the demand for high-quality aquatic products [77]. However, such innovations and research are still lacking in East African countries. Strengthening research and innovations in feed formulations is crucial to effectively meet the specific dietary requirements of different fish species and life stages [78]. This can lead to improved growth rates, feed conversion efficiencies, and the overall health and well-being of farmed fish. Furthermore, advancements in feed processing technologies can enhance the palatability, stability, and shelf-life of fish feeds, ensuring they retain their nutritional value during storage and transportation [45]. Moreover, R&D efforts can also focus on improving feed quality control measures to ensure consistency and safety. By developing robust quality assurance protocols and testing methods, it becomes possible to verify the nutritional content and safety of fish feeds accurately [79]. This instills confidence among fish farmers and feed manufacturers, promoting trust and reliability within the industry.

### 5.3. Integration of Digital Technologies

The adoption of digital technologies presents a significant opportunity to improve the efficiency and sustainability of fish feed production and management in East Africa. Precision farming tools, such as automated feeders and real-time water quality monitoring systems, can optimize feed usage, reduce waste, and enhance fish growth rates [80]. These technologies enable farmers to make data-driven decisions, leading to more efficient feed management practices and improved overall farm productivity. One example is the use of precision farming tools that allow for the precise delivery of feed based on the specific needs of the fish at different growth stages [81]. This method ensures that the fish receive the optimal amount of nutrients, reducing both overfeeding and underfeeding, which are common issues in traditional feeding practices. The implementation of automated feeding systems equipped with sensors and control units can significantly reduce labor costs and improve FCRs.

Additionally, blockchain technology can be employed to enhance traceability in the fish feed supply chain. Blockchain systems provide a transparent and immutable record of feed production, from raw material sourcing to final feed delivery [82]. This traceability ensures the authenticity and quality of feed, thereby increasing farmer confidence in feed products and allowing for better regulatory compliance. For instance, Bumble Bee Foods and SAP's development of a blockchain-based system in 2019 to track and trace fresh fish from the source to the end consumer [83]. Another example is OpenSC, an online blockchain platform launched by WWF Australia and BCG Digital Ventures, which integrates IoT-enabled devices and machine learning classification techniques to track fish throughout the supply chain [84]. These initiatives demonstrate how blockchain technology can improve transparency and accountability if implemented in the fish feed sector. Furthermore, mobile applications and online platforms can facilitate knowledge sharing and training among fish farmers. These digital tools can provide farmers with access to best practices, troubleshooting tips, and real-time advisory services from experts. In Kenya, the use of a mobile app called AquaRech has revolutionized how farmers access information and manage their aquaculture operations, resulting in increased productivity and reduced losses [85].

### 5.4. Policy Influencing and Recommendations for Regulatory Reform and Support

Import taxes on fish feeds and feed ingredients present a considerable obstacle to the development of aquaculture within the EAC, especially given the lack of locally produced high-quality starter and grow-out feeds. To promote the growth of the aquaculture sector, it is crucial to encourage EAC governments to consider temporarily reducing import duties on high-quality fish feeds [49]. This reduction in import tariffs would alleviate the financial burden on fish farmers and promote the accessibility of nutritious feeds necessary for optimal fish growth and development. By reducing import duties on high-quality fish feeds, EAC governments can incentivize the adoption of best practices in aquaculture production. Access to affordable and quality feeds is crucial for achieving desirable growth rates, improving feed conversion efficiency, and ensuring farmed fish's overall health and productivity. Moreover, such measures can stimulate investment in the aquaculture value chain, including feed manufacturing, thereby enhancing the self-sufficiency and competitiveness of the regional aquaculture industry [49].

**Figure 4:**
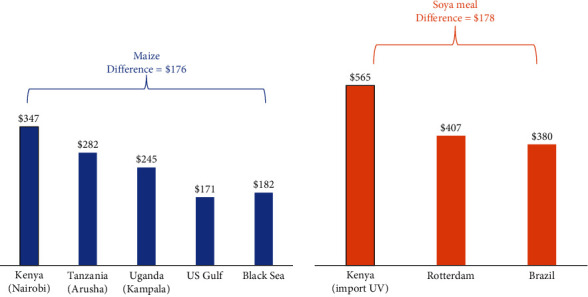
Price of critical feed raw materials [50].

Advocating for temporary reductions in import duties is consistent with broader efforts to enhance food security, economic development, and sustainability within the EAC. By making quality feeds more affordable, governments can bolster the aquaculture sector as a sustainable source of protein and income for communities across East Africa. An analysis by Njagi [50] highlights significant price variations for maize and soya meal across different markets, including Kenya, the East African region, and the global market. For example, maize prices in Tanzania were $65/tonne (19%) lower than in Kenya, and in Uganda, they were $102/tonne (29%) lower. If policymakers approved a duty waiver for globally sourced maize, prices from the Black Sea and the United States would be substantially lower, by $165/tonne (48%) and $176/tonne (51%), respectively, compared to Kenya (Figure 4). Similarly, soya meal from Brazil was 28% cheaper than the imported price in Kenya, while prices in Rotterdam were 32% lower. Transportation costs were estimated at around $45/tonne, with insurance costs accounting for 1.5% of the free on-board price [50].

**Figure 5:**
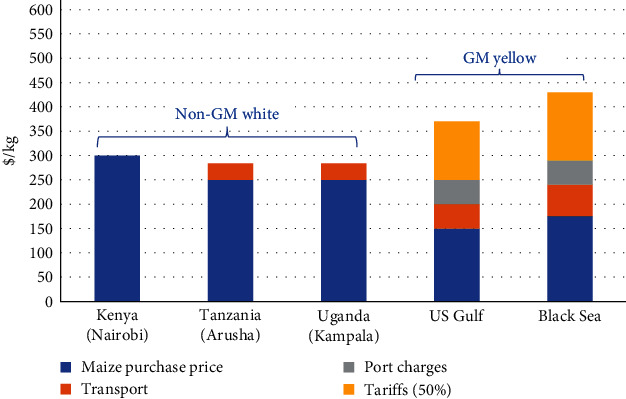
Prices of maize sourced from various markets [50].

Figure 5 illustrates the prices of maize sourced from various markets. Notably, purchasing white maize from regional sources would reduce total expenditure by $15/tonne (5%) after considering transportation and logistics costs. Meanwhile, sourcing yellow GM maize from the Black Sea or the United States would yield even greater savings, with reductions of $17/tonne (6%) and $55/tonne (18%), respectively, assuming a duty waiver. However, it is worth noting that despite the potential cost advantages of importing yellow GM maize, the overall costs, including transportation and port charges, remain higher compared to local maize, even without the duty. This suggests that while global market sourcing may offer cheaper raw materials, the significant transportation and port charges diminish these advantages [50].

### 5.5. Strengthening Quality Control and Standards

Implementing strict quality control measures and regular inspections can ensure that only high-quality fish feed is available in the market, which is crucial for enhancing productivity and profitability in the aquaculture sector. Training and certifying feed producers and suppliers on best practices are essential to maintaining high standards and preventing the distribution of substandard products. This can be supported by developing and enforcing national standards for fish feed quality, which protect farmers from inferior products and promote overall sustainability and growth within the sector. For instance, in Kenya, establishing fish feed standards has significantly improved the quality of available feeds, leading to better feed management practices and cost savings for farmers [21]. Ugachick in Uganda has invested in advanced feed production technologies and strict quality control measures, ensuring the production of high-quality fish feeds that meet both local and international standards [86]. In Tanzania, the introduction of stringent quality control measures has been critical for development, emphasizing the importance of technical skills in feed production and the necessity of adequate standards to ensure market access to high-quality feeds [47]. Additionally, the collaboration between the LVFO and the EAC to develop the “Regional Guidelines for the Certification of Fish Seed and Fish Feed” has been a significant step toward harmonizing quality standards across the region, ensuring consistency and reliability in fish feed production [57]. These initiatives illustrate the feasibility and effectiveness of implementing strict quality control and certification processes in the fish feed sector, leading to enhanced feed quality, increased farmer confidence, and overall sector growth.

### 5.6. Human Capacity Development and Extension Services

Several factors, including inadequate research capacity, limited expertise, and the lack of robust formulation programs and feed models, make effectively utilizing available technology to formulate optimal diets in aquaculture challenging. These issues often result in the use of basic and underperforming feeds. Additionally, the shortage of skilled fish farmers proficient in feed management exacerbates the industry's challenges. Insufficient knowledge of advanced feed formulation techniques and the specific nutritional requirements of different fish species significantly affects production output [87].

To tackle these challenges, EAC governments must prioritize capacity-building initiatives for aquaculture extension workers and fish farmer associations. This involves investing in training programs to enhance the technological proficiency of local fish feed manufacturers and promote the development of high-quality, affordable feeds [88]. Integrating practical fish feed formulation training into university and technical college curricula can produce professionals with hands-on experience, further strengthening the industry's expertise base. Moreover, leveraging information and communication technologies (ICTs) such as radio, video, and smartphone applications offers a cost-effective means of disseminating information and providing ongoing support to fish farmers. These platforms can deliver training materials, share best practices, and collect data to monitor and improve productivity levels in aquaculture [87].

### 5.7. Policy and Public Investment Support

In many East African countries, aquaculture is seen as a crucial strategy for improving food and nutrition security while creating jobs. However, despite established policies and strategies for fish feed development, these initiatives often suffer from inadequate government funding and poor implementation [89]. To further encourage private sector investment and ensure industry sustainability, East African countries need to strengthen policy support, enforce regulations, invest in infrastructure, and support institutional innovations.

Governments must provide robust policy frameworks and high-level backing for the aquaculture sector. Implementing and enforcing regulations are essential to maintaining industry standards. Significant investment in infrastructure is necessary to support the aquaculture industry, and encouraging institutional innovations can help address existing challenges. Notably, prior to 2023, Uganda lacked a specific policy regulating animal feeds, underscoring the need for continued policy development and implementation. The 11^th^ Parliament of Uganda enacted the Animal Feeds Bill of 2023 to regulate the production, storage, importation, exportation, and marketing of animal feeds. According to the bill, anyone wishing to engage in the production, storage, and sale of animal feeds must apply for a license, which will be reviewed within 3 months [90].

Supportive policies might include streamlining business processes, reducing taxes to encourage business growth, and lowering import duties on inputs such as raw materials and equipment essential for local fish feed production [87]. In East Africa, high transportation costs and unreliable electricity supply present significant challenges to attracting and maintaining private sector investment and developing aquaculture value chains. Therefore, investment is crucial for infrastructure development, including road construction and maintenance, transportation systems, and electricity supply, to ensure the continuous operation of the feed industry and meet local demand [87]. Despite the regional government's commitment to improving quality control, there remains a noticeable lack of sufficient capacity.

### 5.8. Sector Institutions and Industry Associations

Establishing a well-funded and authorized aquaculture feed association is crucial to address the institutional gaps caused by the liberalization of the fish feed sector in East Africa. This association, managed by the private sector with government oversight, would play a key role in addressing the various challenges faced by the fish feed industry [91]. Such an organization would be responsible for drafting legislation and regulations, setting membership criteria with enforceable conditions, implementing sector-wide self-regulation and HACCP systems, conducting audits, enforcing standards and regulations, and collecting fees to finance its operations. By consolidating policy, regulatory, and sector development functions, this association would ensure representation from all major stakeholders, including raw material suppliers, premix suppliers, and feed manufacturers.

Leading fish feed manufacturers must collaborate to create the necessary structure and legislation to advance the feed sector in their countries. They should provide strong leadership and financial support and establish a well-equipped secretariat staffed with skilled personnel. The EAC has established EAS 973:2019, Compounded fish feeds—Specification, which outlines requirements, sampling methods, and tests for compounded fish feeds used in aquaculture, specifically for tilapia and catfish. However, feed industry organizations are often underfunded and lack resources, limiting their effectiveness. There is a critical need for a robust institution to govern the industry, covering aspects such as policy development, regulatory frameworks, GMP, quality assurance/control (QA/QC), auditing, feed quality control, training, and investment. With adequate funding and government support, an industry association could effectively engage in self-regulation and significantly contribute to the development of the animal feed sector [92].

### 5.9. Training and Awareness Creation for the Feed Providers

Investing in innovation, knowledge, and skills for commercial feed production is a crucial strategic intervention for the East African feed sector. The region offers various aquaculture training programs at certificate, diploma, and degree levels, designed to equip individuals with the necessary knowledge and skills for the growing aquaculture industry. Notable institutions providing these programs include the University of Nairobi, Maseno University, University of Eldoret, and Ramogi Institute of Advanced Technology in Kenya, Makerere University in Uganda, Sokoine University of Agriculture, University of Dar es Salaam, and University of Dodoma in Tanzania. These institutions offer theoretical and practical training in various aspects of aquaculture, such as breeding, production, and management. However, a significant bottleneck remains the lack of an effective institutional environment for training staff across the fish feed industry [93]. A notable disparity exists between the demand for skilled labor in the sector and the availability of suitable training courses and modules. To address this gap, it is advisable to benchmark the East African feed industry against countries with well-developed feed sectors and tailor the regulatory guidelines and systems accordingly. Countries like South Africa offer valuable insights that could be customized and implemented to meet the specific needs of East Africa [94].

Furthermore, developing innovative approaches and enhancing technical skills are paramount for advancing the competitiveness and sustainability of the East African feed sector. This includes fostering collaboration between industry stakeholders, academia, and government agencies to establish training programs that address the sector's specific needs [93]. Additionally, leveraging international partnerships and best practices can provide valuable insights and expertise to support regional capacity-building efforts [94].

### 5.10. Improved Storage Facilities

Enhanced storage facilities are essential for maintaining the quality of feeds in aquaculture operations. It is advised to store fish feeds in well-ventilated facilities with controlled temperatures to mitigate the effects of temperature and humidity fluctuations [95]. Protection from pest infestations is also critical to prevent contamination and spoilage of the feeds. Implementing a first-in, first-out approach is crucial for ensuring that older feeds are used before newer ones, thereby reducing the risk of feed deterioration over time. To enhance feed management practices, comprehensive guidelines focusing on storage and handling protocols should be developed and widely shared with farmers [96]. These guidelines will assist farmers in optimizing feed quality and minimizing losses, ultimately contributing to the overall success and sustainability of aquaculture operations.

Examples from other sectors highlight the advantages of improved on-farm storage. For example, a study by Huss et al. [97] demonstrated that better storage technology and training significantly reduced food insecurity during COVID-19 restrictions in treatment households. This underscores the potential of enhanced on-farm storage to mitigate vulnerability to food supply shocks, aiding long-term climate change adaptation and balancing public health protection with food security. Additionally, a study by Brander, Bernauer, and Huss [98] in Tanzania found that providing farming households with hermetic storage bags reduced the proportion of severely food-insecure households by 38% during the lean season and by 20% throughout the entire seasonal cycle. These case examples show that with modern storage technologies and comprehensive guidelines, farmers can optimize feed quality, reduce losses, and enhance the sustainability and productivity of aquaculture operations.

### 5.11. Subsidies and Incentives for Local Feed Production

Governments in Kenya, Uganda, Tanzania, and Rwanda should consider providing subsidies for raw materials and tax incentives to local fish feed producers. These measures would lower production costs, making high-quality feed more affordable for farmers. For instance, Kenya's government's feed subsidy program has positively influenced households' decisions to participate in the improved fish feed market. It has increased private sector demand for enhanced feed [99].

Promoting public–private partnerships (PPPs) to invest in local feed production facilities is crucial for reducing dependency on imported feeds. PPPs can encourage the establishment of local feed manufacturing plants that utilize locally available raw materials. One notable example is the FoodTechAfrica initiative, a PPP comprising 21 companies and universities focused on enhancing food security in East Africa through a fully integrated aquaculture value chain. To tackle the shortage of affordable, high-quality fish feed, FoodTechAfrica partnered with experts like Skretting, Almex, Nutreco, Ottevanger, and Unga [100]. Together, these partners established a fully extruded floating fish feed factory in Nairobi, Kenya. This factory combines Unga's local experience, Ottevanger's advanced technology, and Nutreco's expertise in feed formulation to produce high-quality, affordable fish feed. With an annual production capacity of 5000 tonnes, it is the first facility in East Africa to supply high-quality floating fish feeds, which are essential for the growth of the region's aquaculture sector. This development has enabled fish farmers to increase their output and reduce production costs per kilogram, thus improving the overall sustainability and productivity of the aquaculture industry [100].

In Uganda, the partnership between Ugachick and international investors has led to the establishment of a modern feed mill that utilizes locally sourced ingredients. This initiative has not only enhanced local feed production but also created job opportunities and contributed to the overall growth of the aquaculture sector [101]. This approach has been suggested in Tanzania, where the availability and nutritive value of local feed ingredients such as maize bran, which provides medium–high crude protein content, can be effectively utilized to develop cost-efficient and nutritious fish feeds [46]. Such partnerships can bring in the required technology, capital, and expertise, thus boosting local feed production capabilities.

### 5.12. Enhancing Gender Participation in the Fish Feed Sector

Women play a pivotal role in fish farming and processing in East Africa [102]. Despite their significant contributions, they often face numerous barriers that limit their full participation in the sector. These barriers include limited access to credit, training, and high-quality inputs such as fish feed [102]. One effective strategy to enhance women's participation is improving their access to critical resources and training. Equipping women with the necessary skills and knowledge will empower them to take on more significant roles in the sector, leading to improved productivity and economic empowerment. Access to credit is another significant barrier for women in the fish feed sector. Kenya currently lacks a specific agricultural finance policy, which creates a potential gap in women's access to financial resources for agricultural activities. Establishing such a policy is crucial to ensure that rural households, including women, have access to appropriate and affordable financial services [103]. An agricultural finance policy would help provide demand-driven financial services, with particular provisions aimed at supporting women and youth in the agricultural sector. For instance, microfinance can offer financial products tailored to women in aquaculture. These products can offer low-interest loans and flexible repayment terms, enabling women to invest in high-quality fish feed and other essential inputs [104]. This will lead to an increased number of women-led aquaculture enterprises, which will lead to greater economic independence and enhanced livelihoods for women in the sector.

Addressing social and cultural barriers is also essential for fostering a more inclusive fish feed sector. For instance, in Bungoma County, Kenya, cultural practices do not generally restrict women from participating in aquaculture [105]. However, it was noted that women are expected to wear trousers when they get into the ponds for maintenance or harvesting the fish. This specific requirement highlights the importance of understanding local cultural dynamics when promoting gender participation in aquaculture. Additionally, while men typically do not process or sell fish in the markets, women are predominantly involved in these activities [105]. Such cultural norms must be taken into account to develop strategies that effectively support women's roles in the fish feed sector.

## 6. Conclusion

This review sheds light on the pressing issues and potential solutions within the fish feed industry in East Africa. The sector faces significant challenges, including limited availability and accessibility of quality ingredients, regulatory hurdles, inadequate quality control measures, infrastructure constraints, and a lack of awareness and expertise in feed management and formulation. To address these challenges, the paper has proposed actionable strategies that encompass fostering collaboration to build a robust regional fish feed supply chain, investing in R&D initiatives, advocating for policy reforms and regulatory support, enhancing human capacity through training and extension services, promoting public investment support, strengthening sector institutions and industry associations, and facilitating training and awareness programs for feed providers. Additionally, emphasis is placed on the importance of improving storage facilities to maintain feed quality. Addressing the challenges outlined in this review is crucial for ensuring the sustainability and growth of the aquaculture sector, meeting the rising demand for fish, and contributing to food security and economic development in the region.

## Figures and Tables

**Table 1:** The approximate cost of fish feed (to the farm) [24].

Country feed Rwanda	($/MT) (2021 prices)
Kenya	850–950
Uganda	850–950
Rwanda	1100–1200
Tanzania	1073–1287

*Note*: Feed prices have increased by up to 30% over 2024, owing to the ongoing Russia-Ukraine War and the continued effects of the COVID-19 economic fallout.

**Table 2:** Common fish feed raw materials used by the millers in East Africa [23].

Raw material	Source (locally available/imported)			
	Kenya	Uganda	Tanzania	Rwanda
Main plant sources				
Cassava (*Manihot esculenta*) flour	Locally available and also imported from Thailand, India, Germany, South Africa, and the United States	Locally available and also imports from Kenya, Monaco, and Rwanda	Locally available and also imported from India, Nigeria, and Rwanda	Uganda
Cotton (*Gossypium* spp.) seed cake	Imported from Tanzania, Uganda, and Zambia	Tanzania	Locally available	Uganda
Cotton (*Gossypium* ssp.) meal	Imported from Tanzania and Uganda	Locally available	Locally available	Imported from Burundi
Maize (*Zea mays*) germ	100% imported from India, Uganda, and Tanzania	Locally available and imports from South Africa, Rwanda, and the United Arab Emirates	Locally available and also imported from India	Locally available
Maize (*Z. mays*) grain	80% obtained locally, 20% imported from Uganda, India, and Brazil	Locally available and Imports from Tanzania, South Africa, and China	Locally available and additional imported from Zambia, India, and the United Arab Emirates	Locally available; additional imported from Uganda, Belgium, and France
Rice (*Oryza sativa*) bran	Locally available and additional imported from Tanzania, Uganda, and India	Locally available; additional imported from India, Tanzania, and Rwanda	Locally available and additional imported from India and Pakistan	Locally available and additional imported from India
Rice (*O. sativa*) polish	Imported from Tanzania, Uganda, India, and Pakistan	Locally available; additional imported from Tanzania, Pakistan, and the United Arab Emirates	Locally available; additional imported from India, Pakistan, and China	Imported from Uganda, Belgium, and India
Soya meal	Imported from Zambia, Malawi, Uganda, and Tanzania	Locally available; additional imported from Zambia, Malawi, and India	Locally available and additional imported from Zambia, Malawi, and Mali	Imported from Uganda
Sunflower (*Helianthus annuus*) cake	Imported from Tanzania, Uganda, Burundi, and India	Locally available; additional imported from Tanzania, the United Arab Emirates, and India	Locally available and additional imported from India	Imported from Uganda
Wheat (*Triticum aestivum*) grain	Locally available, imported from Uganda, India, and the United Kingdom	Locally available; additional imported from Russia, Argentina, and Ukraine	Imported from India	Imported from Belgium
Wheat (*T. aestivum*) Pollard	Imported from Uganda, Rwanda, and Ethiopia	Locally available; additional imported from Rwanda, Burundi	Imported from Turkey, India, and South Africa	Locally available
Main animal sources				
Fish meal (*R. argentea*)	Locally available; additional imported from Tanzania, Uganda, China, and India	Locally available; additional imported from Kenya, Nigeria, and Zambia	Locally available; additional imported from Zambia, South Africa, and China	Imported from Tanzania, Kenya, Uganda, China, India, and Zambia
Freshwater shrimp (*Caridina nilotica*)	Locally available; additional imported from Tanzania and India	Locally available; additional imported from the United Kingdom	Locally available; additional imported from Indonesia	—
Bone meal	Locally available; additional imported from Tanzania	Locally available	Locally available	—
Other raw materials				
Vitamin and mineral premixes	Imported from South Africa, the United Kingdom, and India	Imported from Turkey, India, and Namibia	Imported from India	Mineral premix from Turkey
Amino acids	Imported from South Africa, China, India, Germany, and the Netherlands	Imported from India, China, and Ireland	Imported from India, Germany, and China	Imported from Belgium, Uganda, and India

## Data Availability

Data sharing does not apply to this article as no new data are created or analyzed in this study.
